# Circulating endothelial cells transiently increase in peripheral blood after kidney transplantation

**DOI:** 10.1038/s41598-021-88411-4

**Published:** 2021-04-26

**Authors:** H. Tejeda-Mora, J. G. H. P. Verhoeven, W. Verschoor, K. Boer, D. A. Hesselink, M. W. F. van den Hoogen, L. J. W. van der Laan, C. C. Baan, R. C. Minnee, M. J. Hoogduijn

**Affiliations:** 1 Department of Internal Medicine, Nephrology and Transplantation, Erasmus MC, University Medical Center, Rotterdam, The Netherlands; 2 Department of Surgery, Erasmus MC, University Medical Center, Rotterdam, The Netherlands

**Keywords:** Biomarkers, Medical research, Nephrology

## Abstract

The diagnosis of kidney allograft rejection is based on late histological and clinical markers. Early, specific and minimally-invasive biomarkers may improve rejection diagnosis. Endothelial cells (EC) are one of the earliest targets in kidney transplant rejection. We investigated whether circulating EC (cEC) could serve as an earlier and less invasive biomarker for allograft rejection. Blood was collected from a cohort of 51 kidney transplant recipients before and at multiple timepoints after transplantation, including during a for cause biopsy. The number and phenotype of EC was assessed by flow-cytometric analysis. Unbiased selection of EC was done using principal component (PCA) analysis. Paired analysis revealed a transient cEC increase of 2.1-fold on the third day post-transplant, recovering to preoperative levels at seventh day post-transplant and onwards. Analysis of HLA subtype demonstrated that cEC mainly originate from the recipient. cEC levels were not associated with allograft rejection, allograft function or other allograft pathologies. However, cEC in patients with allograft rejection and increased levels of cEC showed elevated levels of KIM-1 (kidney injury marker-1). These findings indicate that cEC numbers and phenotype are affected after kidney transplantation but may not improve rejection diagnosis.

## Introduction

Kidney transplantation stands as the optimal therapeutic procedure for patients affected by end-stage renal disease. Improvements in immunosuppressive therapy have prolonged short-term graft survival and helped in decreasing rejection^1^. To date, renal function after transplantation is mainly assessed by serum creatinine concentration, urinary protein excretion and renal transplant biopsy in case rejection is suspected. Nonetheless, these approaches have several drawbacks. Serum creatinine levels increase late after injury and are non-specific for the type of injury^2^. Additionally, creatinine levels are not very useful to predict the progression of chronic injury^3,4^. Biopsies provide insight in the status of kidney transplants and cause of renal allograft function decline. However, biopsies cannot be performed continuously due their invasive and potentially harmful character, and the diagnosis can be missed as a result of sampling error (the tissue specimen typically exemplify about 0.01% of the volume of the organ^5–7^. Therefore, the development of minimally invasive, reliable and predictive biomarkers for early diagnosis and monitoring of kidney transplant health is essential for improving transplant outcome^8–11^.

Endothelial cells (EC) cover the inner side of all vasculature in tissues. It is widely accepted that ECs posses unique phenotypic, functional, and angiocrine characteristics^12–14^. EC have malleable cellular features that allow them to respond to normal physiological stress and to promote tissue homeostasis. Upon injuries or inflammation, EC recruit and activate circulating leukocytes by increasing the expression of adhesion molecules and chemokines. Simultaneously, EC are able to initiate regeneration pathways. The correspondence between their proinflammatory, vasoconstrictive, and regenerative capacities might influence allograft acceptance^15^.

Circulating endothelial cells (cEC) are mature cells held as reliable markers of endothelium derangement. Healthy subjects have low cEC counts, whereas it has been demonstrated that levels increase significantly in conditions associated with vascular damage, as cancer and cardiovascular diseases^16–19^. In the specific case of kidney transplantation, the allograft micro- and macrovasculature is affected by ischemia–reperfusion injury (IRI) during the transplant procedure, and later by acute rejection episodes leading to endothelial injury and EC shedding^15,18^ side with renal fibrosis and loss of renal function. Thus, the adaptive functions and characteristics of cEC may position them as a potential biomarkers for organ viability in organ transplantation.

EC identification can be performed before and after kidney transplantation. We hypothesized that upon injury, cells within the capillaries are likely to get released from the graft. After organ transplantation, characterization of EC from peripheral blood represents a valuable methodology to avoid invasive diagnostic procedures. Furthermore, understanding the phenotypical determinants of cEC could aid in clinical decision making, developing efficacious strategies for organ repair, and improving long-term graft survival after transplantation.

In the present study we examined whether injury induced by the transplant procedure and rejection episodes is associated with release of EC, with elevated cEC and with phenotypic changes in cEC. To this end, we measured EC numbers in 3 deceased donor kidney machine perfusion perfusates and in the blood of 51 kidney transplant recipients. We additionally examined the cEC phenotype to evaluate their potential as biomarker for transplant status diagnosis. Lastly, we investigated the cEC origin and made correlations with clinical variables related with this biomarker and its relevance in the clinical setting for renal transplantation.

## Results

### Endothelial cells are present in kidney perfusion solution

**Figure 1:**
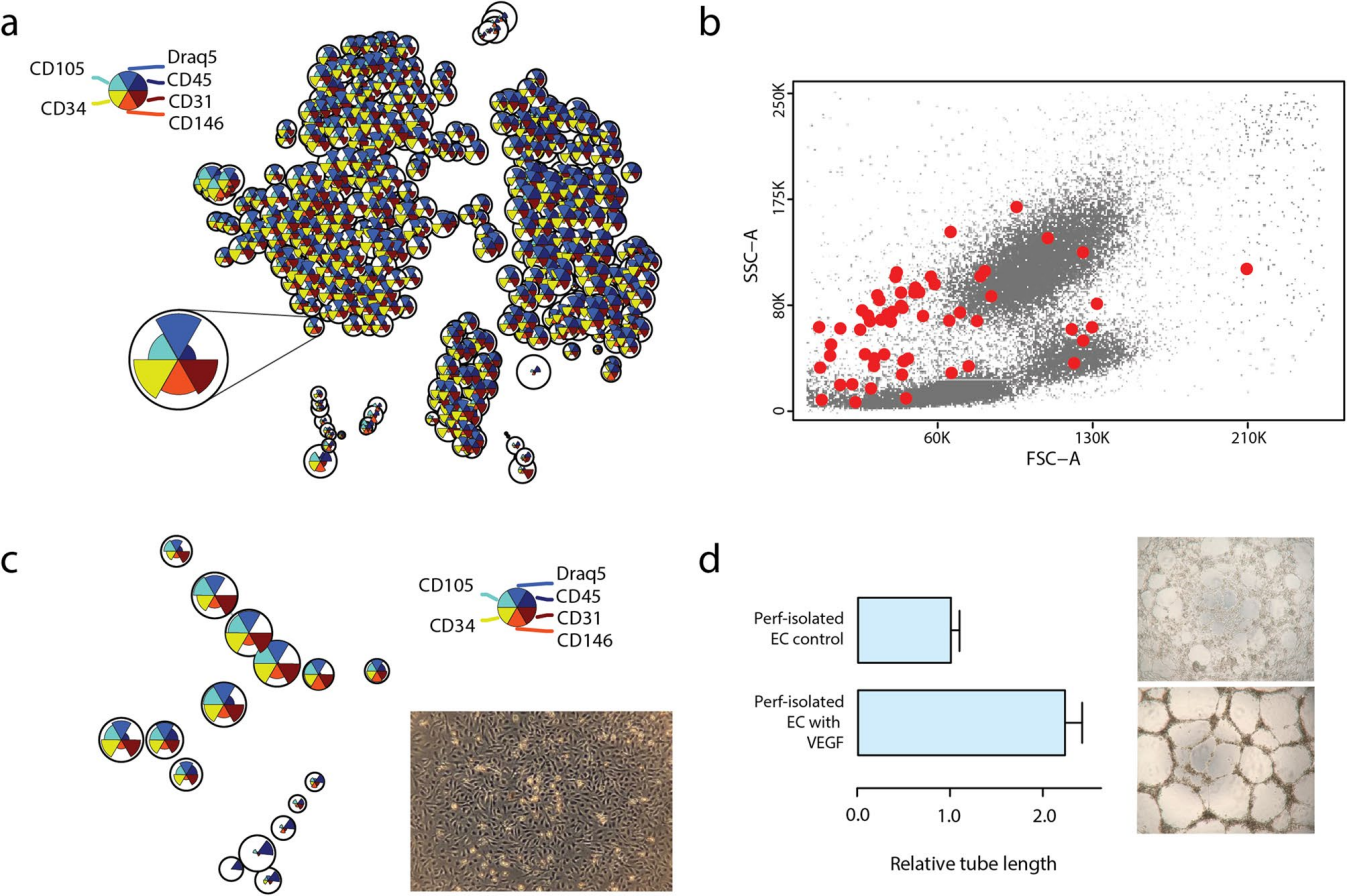
Identification of EC in kidney perfusates. Perfusate cells were stained for CD45, CD31, CD34, CD146, CD105, and Draq5. EC were identified by non-biased clustering. **(a)** t-SNE representation of cell clusters; cluster size indicates amount of cells; pie charts inside clusters depict the mean fluorescence intensities (MFIs) of the indicated markers in the cluster. The EC cluster is magnified. **(b)** Dot plot depicting cells in perfusion solution, EC are marked with red dots. **(c)** Cells isolated from perfusates on the basis of their morphology and subsequently expanded showed EC immunophenotype and typical cobblestone morphology. **(d)** Branching network forming capacity upon stimulation with VEGF. The tube length in three randomly chosen fields from each well was measured. Data is expressed as mean of 4 experiments ± SEM.

Endothelial cells (EC) from deceased donor kidneys were identified in machine perfusion perfusates by flow cytometry using a non-linear generalization of principal component analysis (PCA) and unsupervised clustering (Fig. 1a). 500 clusters were used to classify cells, based on data set variance, and robustness of detected endothelial cell clusters. Cluster criteria selection was set up to determine EC containing clusters (CD45^-^, CD31^+^, CD34^+^, CD146^+^, CD105^+^, DRAQ5^+^). For most samples one cluster contained all EC (Fig. 1a). The amount of EC present in perfusates was 0.57 cells/ml on average and varied between donors from 0.23 to 1.03 cells/ml of perfusate (n = 3). The EC found recorded differences in size and granularity (Fig. 1b). To confirm EC identity, we cultured the cells present in kidney perfusates. The majority of the selected and expanded cells showed a similar expression profile compared to the non-cultured EC in perfusates (Fig. 1c). An angiogenesis assay was carried out to provide evidence that the isolated cells possessed EC functional properties. Culture expanded EC from the perfusate recorded nearly 2.5-fold increase in tube length after stimulation with VEGF, confirming they possessed EC functional properties (Fig. 1d). These results indicate that donor kidneys release EC before transplantation.

### Kidney transplant recipients show an increase in cEC number shortly after transplantation

**Figure 2:**
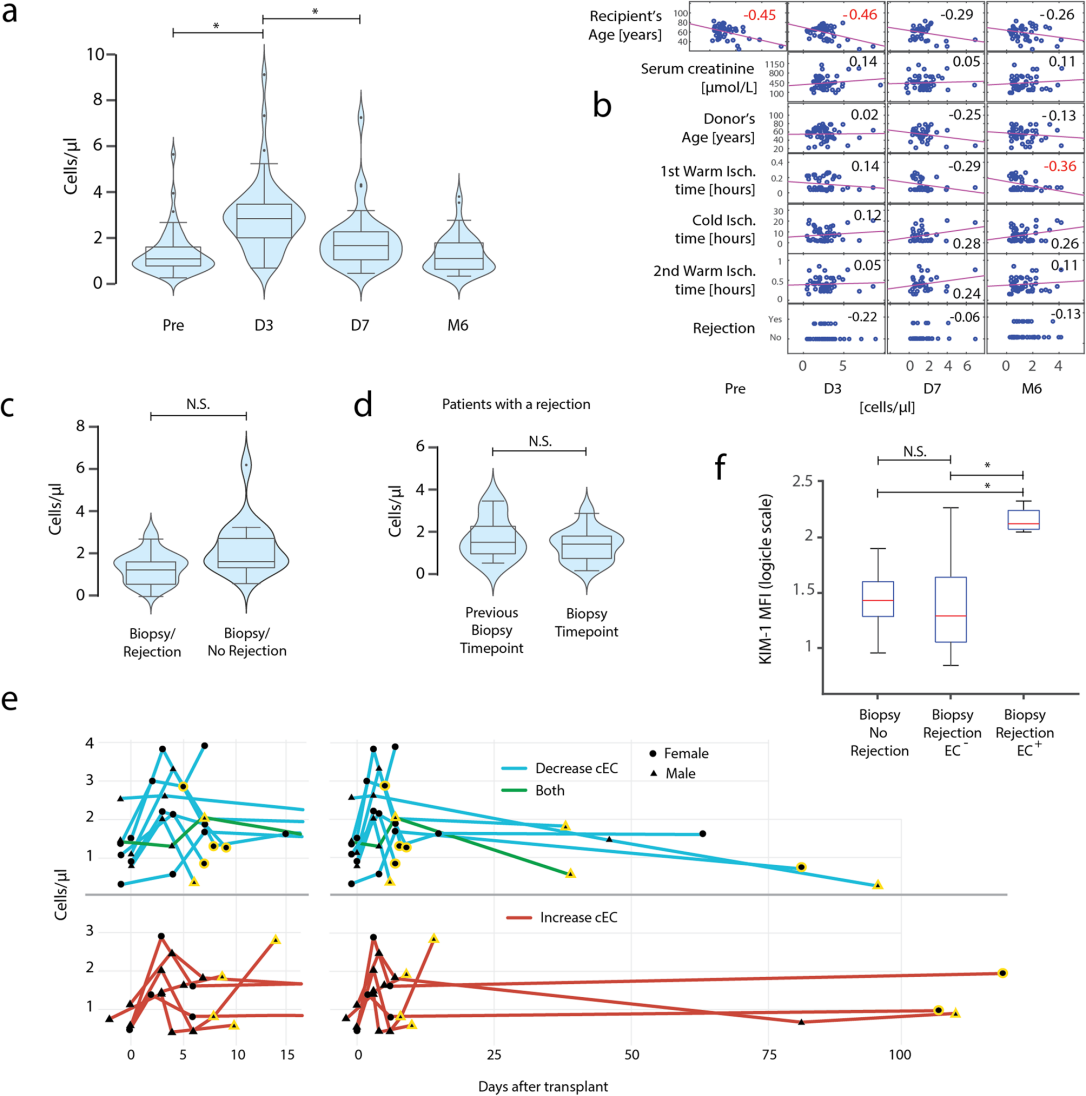
EC are detected in venous blood of kidney transplant recipients. **(a)** cEC numbers in patients for all measured time points. **(b)** Scatter plots with correlations between numbers of cEC in measured time points and patient and donor variables. The displayed Pearson’s linear correlation coefficients indicate the slopes of the least-squares reference lines in the scatter plots. Logistic regression between numbers of EC in measured time points and rejection. The displayed logistic coefficients indicate the change in the log odds of having a rejection. Coefficients marked in red show which pairs of variables have correlations significantly different from zero (p < 0.05). **(c)** Numbers of cEC at the time of biopsy taking in patients with and without a rejection episode. **(d)** Numbers of cEC in patients with a rejection episode at the time of biopsy taking compared to its respective previous measured time point. **(e)** cEC levels in patients with biopsy-proven rejection, classified by the change in cEC concentration compared to the previous measured timepoint. Time points where biopsies were performed are highlighted in yellow. Magnifications on the first 15 days after transplantation are shown at the left of the original plot. **(f)** Expression of KIM-1 (MFI values) in cEC of patients without rejection, with a rejection but without an increase in cEC, and with a rejection with an increase in cEC for the different biopsy outcomes in patients. Data are expressed as the median together with the 25th and 75th percentiles. In **(a)**, n = 50 for Pre, n = 49 for D2, n = 34 for D7, and n = 46 for M6; in **(c)** n = 17 biopsies with rejection, n = 25 biopsies with no rejection; in **(d)** n = 17; in **(f)** n = 25 biopsies with no rejection, n = 9 biopsies with rejection and decrease, n = 8 biopsies with rejection and increase in cEC; (* p. < 0.001). In a) one-way ANOVA with Student’s post hoc pair-to-pair test; in **(c,d,f)** Mann–Whitney U-test was used.

We went on examining whether cEC can be detected in patients after transplantation. We quantified and phenotyped EC in the blood of 51 kidney transplant recipients before and after transplantation. Of these, n = 46 completed the 6-month follow-up. Two patients died before month 6, two had a transplantectomy and one dropped out the study for another reason. These missing patient samples were pairwise deleted from all performed analyses. The same clustering procedure used in perfusates was followed to detect EC in blood. We detected 1.28 ± 0.96 cEC/µl before transplantation. cEC numbers were significantly increased 3 days after transplantation (2.05 fold on average, p < 0.001) (Fig. 2a). At day 7 cEC numbers returned close to preoperative levels (p < 0.001, compared to day 3); 6 months after transplant cEC numbers were similar to pre-transplantation levels (1.29 ± 0.91 cEC/µl). We checked whether the number of cEC in recipients could be indicative of the status of the transplantation and an early biomarker for kidney injury and rejection. Therefore, we compared cEC numbers with patient and donor parameters such as sex, age, creatinine concentration, delayed graft function (DGF), cause of end-stage renal disease, preservation time, donor type, rejection type, dialysis type, induction therapy and other clinical parameters shown in Table 1 (Fig. 2b, Supplementary Fig. S1-2 online). Patient age and the length of the first warm ischemia time were the only substantial and negatively correlated variables with cEC numbers across time points.

**Table 1:** Overview of recipient and donor characteristics.

Kidney transplants		
Total included	n	51
Recipient age	Range	24–83
Sex	F, M	24,27
Race	C, As, B, Ar	41,5,3,2
Rejection	n (TCMR, aABMR, ACR)	15 out of 51 (29.4%) (9,4,2)
Dialysis (type)	n (HD,PD)	34 out of 51 (25,9)
Induction therapy	Bas, rATG, Alem	46, 1, 4
Donor type	LRD, LURD, DCD, DBD	8,15,19,9
Donor age	Range	22–79
Donor sex	F,M	23,28
Perfusion type (for DCD and DBD donors)	HMP, NMP, SCS	21,1,6

Recipients; median age and range, distribution of males and females, race (*C* Caucasian, *As* Asian, *B* Black, *Ar* Arab), rejection type (*TCMR* T cell-mediated rejection, *aABMR* acute antibody-mediated rejection, *ACR* borderline acute cellular rejection), dialysis type (*HD* hemodialysis, *PD* peritoneal dialysis), and induction therapy (*Bax* Basixilimab, *Alem* Alemtuzumab). Donors; donor type (*LRD* living related donor, *LURD* living unrelated donor, *DCD* donation after cardiac death, *DBD* donation after brain death), median age and range, distribution of males and females, and perfusion type (*HMP* hypothermic machine perfusion, *NMP *normothermic machine perfusion, *SCS* static cold storage).

### Rejection events do not influence the number of cEC

Patients with a biopsy-proven rejection (n = 15) recorded heterogeneous cEC concentrations (p = 0.169), comparable to patients who got a biopsy but in whom another diagnosis than rejection was made (n = 11) (Fig. 2c). Similarly, no significant change was observed when cEC levels at the time of rejection were compared to the time point prior to the rejection event (n = 15; p = 0.281) (Fig. 2d). Rejectors were classified regarding their change in cEC during rejection events compared to the previous measured time point (Fig. 2e, Supplementary Table S1 online). Interestingly, rejectors with an increase in cEC reported an increase in KIM-1, while rejectors without an increase in cEC did not show elevated KIM-1 expression. (Fig. 2f).

### Progenitor phenotype of cEC relates to recipient age

**Figure 3:**
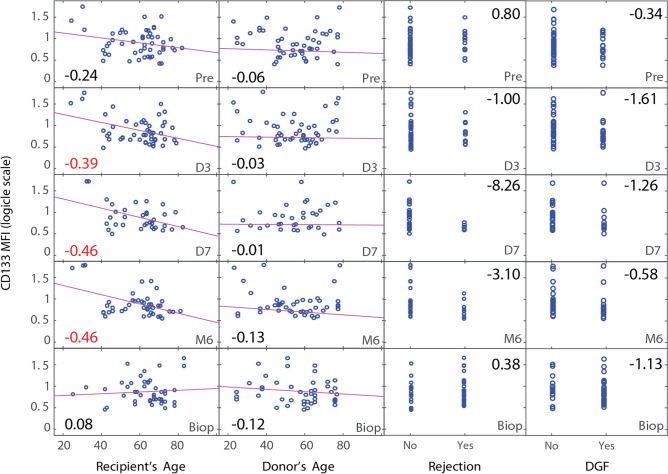
Expression of CD133 in cEC of kidney transplant patients. Correlations between recipient and donor age, and CD133 MFI expression in cEC before and after transplantation. The displayed Pearson’s linear correlation coefficients indicate the slopes of the least-squares reference lines in the scatter plots. The two columns on the right show the logistic regression between CD133 in cEC and rejection and DGF. Patients with DGF were such patients who needed renal replacement therapy within the first seven days after transplantation. The displayed logistic coefficients indicate the change in the log odds of having a rejection. Coefficients marked in red are significant (p < 0.05).

Within our EC detection panel, we included the endothelial progenitor cell marker CD133 to get insight into the frequency of cEC with potential regenerative characteristics (Supplementary Fig. S3 online). No significant relationship was found between the expression of CD133 on cEC and graft rejection nor DGF (Fig. 3). Similarly, as with the number of cEC, we found a negative correlation between the age of the recipient and the MFI expression of CD133. The age of the donor did not have an influence on the CD133 expression on cEC (Fig. 3).

### cEC are of mixed donor and recipient origin

**Figure 4:**
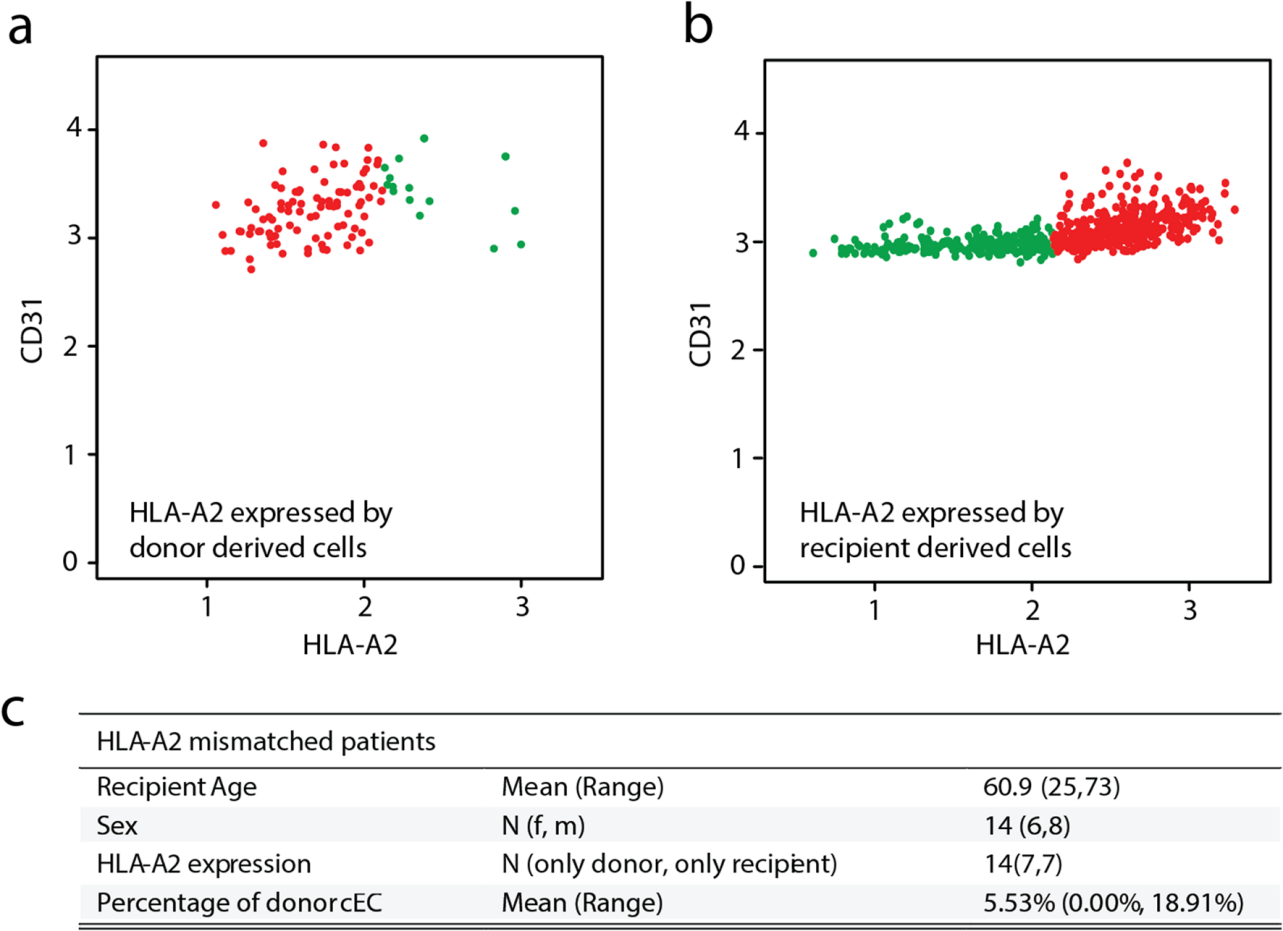
cEC are derived from both the recipient and the donor kidney in transplant patients. Dot plots showing donor and recipient EC in peripheral blood of kidney transplant patient 3 days after transplant; HLA-A2 cEC are shown in green. In **(a)**, sample from a recipient where only the donor expressed HLA-A2; in **(b)**, recipient expressed HLA-A2, but not the donor. **(c)** Overview of recipient and donor characteristics.

After corroborating the presence of cEC in patients we investigated cEC origin. We hypothesized that the increase in cEC at day 3 after transplant arose from the kidney, supported by the finding of EC in perfusates. We sought kidney transplant donor-recipient couples with an HLA-A2 mismatch (Supplementary Table S2). We included 14 patients; 7 kidney transplant recipients whose donor expressed HLA-A2, and 7 kidney transplant recipients who expressed HLA-A2 but not the donor, and examined HLA-A2 expression on cEC by flow cytometry. Unbiased clustering was followed to identify the two possible cEC populations (Supplementary Fig. S4 online). We detected donor kidney derived cEC in 13 out of 14 patients (Fig. 4a,b). The percentage of donor-derived cEC found in patients was in the range of 0.95–18.91% of the total cEC (Fig. 4c). This demonstrates that the origin of cEC is mixed whereas the majority of cEC is recipient derived.

## Discussion

The endothelium is one of the earliest cell types to be affected by ischemia and immunological injury. In the present study we used several markers and an unbiased gating methodology to identify EC in kidney machine perfusion perfusates and in peripheral blood via flow cytometry. Our results demonstrate the presence of kidney transplant-derived EC in machine perfusion solution and suggest that ischemic damage in donor kidneys triggers EC detachment. We found that cEC are mainly of recipient origin and we observed no association between cEC number fluctuations after kidney transplantation and transplant outcome.

Machine perfusion is increasingly used to preserve kidneys and it provides an excellent opportunity to examine the kidney before transplantation^20,21^. In the present study, we confirmed that grafts release EC after perfusion. We propose that EC are partly released from the graft through manipulation during the transplantation procedure.

We observed a significant increase in cEC numbers shortly after kidney transplantation. Baseline levels of cEC concentration were recovered for most patients after one week and completely returned to preoperative levels after 6 months. This is a novel finding as other studies have just measured the concentration of cEC at a single time point^22–25^. The dynamics observed in the level of cEC reflects the disturbance caused to the graft and recipient’s endothelium shortly after the transplantation procedure. Nevertheless, the injury caused to the graft during a rejection event had no direct influence in the amount of cEC. We believe changes in cEC concentrations depend on the severity of vascular stress. While a transplantation is a highly invasive procedure with a direct impact on the donor kidney and recipient endothelium due to the procedure itself and donor effects, a rejection event is caused by immunological factors at the interplay of other responses that trigger inflammation and aberrant vasculature responses^26,27^. The later ones appear not to cause substantial EC shedding.

The previously described cEC concentration fluctuation was also observed in 11 out of the 15 patients who had biopsy-proven rejections before the 6-month time point. Furthermore, cEC levels were not associated with different types of rejection nor the vascular damage assessed by histological evaluation. Interestingly, we observed that during rejection events with an increase in cEC numbers, cEC elicited a higher expression of KIM-1. It is known that the allograft fate is associated with the degree of the rejection^5^. Nevertheless, the increase in cEC concentration and its KIM-1 expression were not connected with the type of rejection. Although acute rejection leads to vascular damage, we hypothesize the early treatment for rejection added complexity to detect changes in cEC concentrations and its phenotype after transplantation.

Beside the correlation between age and the amount of cEC previously reported in renal transplant recipients and healthy subjects^23^, we identified other patient and donor variables that influenced cEC numbers at different stages of transplantation. Shorter warm ischemia times significantly increased the number of cEC 6 months after transplantation. Since we observed no significant correlation between the other time points or ischemia times, we ought to compare the amount of cEC with the total ischemia time, where no significant correlation was observed (Supplementary Fig. S5 online). Moreover, we found that patients with higher mismatches in HLA (classes A and B), in addition to having a higher chance for a rejection episode^28,29^, also reported a higher cEC number during later time points (Supplementary Fig. S1 online). This suggests that ongoing immune responses against the donor organ may be a factor that stimulates EC release over time. Therefore, we hypothesized that donor cEC could be used as a quality metric after kidney transplantation. Contrary to expectation, the concentration of donor related cEC found within the recipient was not indicative for recipient status. In the present study the methodology used to identify donor/recipient cEC was solely based on the detection of HLA-A2 mismatches. In order to potentiate these findings inclusion of patients with different HLA mismatches is necessary. Nevertheless, this result added evidence that EC shedding is not solely influenced by a rejection event, and that further research is needed to determine the main causes of cEC levels increase.

The use of more sensitive markers for EC characterization in blood may lead to a better classification of EC subpopulations in patients. We included within our EC flow cytometry panel the EPC marker CD133. EPC are involved in repair of various types of vascularized tissues and have shown to be a promising repair tool in animal experiments and clinical trials^30–32^. In our study low concentrations of circulating endothelial progenitor cells (cEPC) were only found in young kidney recipients, whereas the donor age did not influence the cEPC concentration in the recipient. Similar results were observed in a mouse model, where EPC mobilization after injury was more robust in the younger animals^33^. As cEPC are a very rare population, cEPC detection by antigens is challenging and may compel the use of different approaches and techniques for EPC identification^34,35^. This would enhance the sensitivity of the assay and give better insight into ongoing organ repair.

Our findings show that donor kidneys release EC during the transplantation procedure. In addition to this initial EC release, recipients show an increase in cEC at day 3 post transplantation. Contrary to reported for other vascular damage pathologies^16,36–38^, injury derived from acute rejection episodes did not influence cEC levels. The identified cEC included donor derived cells even after 6 months post transplantation. These data shows that the amount of donor derived cEC in kidney transplant recipients is lower than the ones reported previously^39^. Our approach for measuring cEC in kidney transplant recipients shows that the endothelium undergoes major changes during early stages of transplantation. On the whole, the concentration of cEC, together with cEC phenotype, give an incomplete figure of the transplant status. This approach of cEC characterization will likely prove effective if expanded with markers that could identify the status of cEC and if those are actively contributing to injury recovery or setting the stage for fibrosis.

## Methods

### Study design and participants

Fifty-one consecutive adult patients (≥ 18 years) who received a kidney transplant were included between March 2019 and August 2019 at the Erasmus MC, Rotterdam, the Netherlands. Written informed consent was obtained from all patients and all living donors before inclusion. All patients could be included, except for patients who also received different organ transplants (i.e. combined transplants). The study was performed in accordance with the declaration of Helsinki (2013) and was approved by the institutional review board of the Erasmus MC (Medical Ethical Review Board number 2018-035).

Donor kidneys in the study included both deceased donor organs and living donor organs (living related donors and living unrelated donors). Kidneys obtained from deceased donors were subjected to either Hypothermic Machine Perfusion (HMP), Normothermic Machine Perfusion (NMP) or Static Cold Storage (SCS).

All patients received induction therapy with either basiliximab (Simulect; Novartis Pharma, Basel, Switzerland), or rATG (Thymoglobulin, Sanofi-Genzyme), or alemtuzumab (Campath, Sanofi-Genzyme, Cambridge, MA). The maintenance immunosuppressive therapy consisted of mycophenolate mofetil (Cellcept; Roche Pharmaceuticals, Basel, Switzerland), glucocorticoids and tacrolimus (Prograft; Astellas Pharma, Leiden, the Netherlands).

### Blood and perfusate collection

Venous blood of kidney transplant recipients was collected in heparinized blood collecting tubes. Blood was collected at the following time points: before kidney transplantation, 2–4 and 6–8 days after transplantation, and 6 months after transplantation. Additional blood samples were collected at the day a clinically-indicated renal transplant biopsy was taken within the first six months after transplantation. Samples were processed between 1 and 24 h after collection. Samples that were not processed within the first two hours after collection, were stored at 4 °C.

UW machine perfusion perfusate samples were collected from kidneys of deceased after circulatory death donors (DCD; n = 3). These samples arose from kidneys that were perfused using hypothermic perfusion. Subsequently, the samples were stored at 4 °C. Perfusates were processed between half an hour and eight hours after collection. Collected volume was 0.1 L. Perfusates, collected from these deceased donor kidneys, are considered as left over material. Therefore, no specific informed consent was necessary from the deceased donors.

### Endothelial cell culturing from perfusates

Kidney perfusates were centrifuged at 800 RCF for 5 min. Later, all cells from kidney perfusate solution were washed in PBS and incubated for 10 min in darkness with FACS lysing solution (BD Biosciences, San Jose CA, USA) to remove red blood cells. The remaining cells were cultured in Endothelial Growth Media (EGM2; Lonza, Walkersville MD, USA). Culture media was refreshed every two days. Potential EC colonies were isolated by mechanically removing colonies with non-EC morphology under the microscope. After expansion, EC identity was confirmed by flow cytometry using the following anti-human antibodies: CD45-PerCP (clone 2D1; BD Biosciences), CD31-PECy7 (clone WM59; BioLegend, San Diego CA, USA), CD34-APC (clone 8G12, BD Biosciences), CD146-AmCyan (clone P1H12; BD Biosciences), CD133-BV421 (clone 7, BioLegend) and CD105-FITC (clone 266; BD Biosciences).

### Angiogenesis 3D gel assay

A tube formation assay was performed to evaluate the angiogenic potential of EC. Geltrex LDEV-Free Reduced Growth Factor Basement Membrane Matrix (Thermo Fisher Scientific—Gibco, Rockford IL, USA) was kept at 4° C overnight prior to the experiment to allow complete thawing. 50 µl Geltrex was added to each well of an ice-cold 96-well plate using cold pipette tips to avoid premature Geltrex solidification. Cells were stimulated with 25 ng/ml of VEGF (R&D Systems, Abingdon, UK). Plates were incubated for 30 min at 37 °C to allow Geltrex solidification. Cells were added to the wells in a concentration of 2 × 10^4^ cells per well. After 6 h, pictures were taken to evaluate the formation of tube-like structures (i.e. angiogenic capacity). The total length of the tubes formed during the assay was measured. Images were analyzed using ImageJ (version 1.52p).

### Circulating endothelial cell identification by flow cytometry

Venous blood samples were centrifuged for 10 min at 1885 RCF. Plasma was exchanged for PBS. Four milliliters of the cell suspension were taken and FACS lysing solution was added in a proportion of 8:1 to induce red-cell lysis. Lysis was performed for 45 min in darkness at room temperature. After lysis, cells were washed twice with PBS + 2% FBS. Cells were resuspended to a final concentration of 10^6^ cells/ml. The following anti-human antibodies were used for identification and phenotyping of cEC: CD45-PerCP, CD31-PECy7, CD34-APC, CD146-AmCyan, CD133-BV421, CD105-FITC. The antibody for kidney injury molecule-1 (KIM-1) CD365-PE (clone 1D12; BioLegend) was used as endothelial injury marker^40,41^. The DNA dye 1,5-bis[2-(dimethylamino)ethyl]amino-4, 8-dihydroxyanthracene-9, 10-dione (DRAQ5; BioStatus Ltd, Shepshed, UK) was used to exclude non-nucleated and polynucleated cells. Antibodies were titrated individually and fluorescence minus one (FMO) controls for CD133 and CD365 were included. Following labeling, cells were washed twice with Facsflow solution (BD Bioscience) before analysis by flow cytometry. Data acquisition was done at a low flow rate (10 µL min^-1^). At least 1.5·10^6^ cells were recorded on BD FACS Canto II (BD Bioscience). Measurements were performed in a blindfolded fashion.

The same procedure was followed with perfusion solutions, with the difference of a shorter lysis time (10 min) due to a lower amount of red blood cells in the perfusion samples.

To distinguish between donor/recipient derived endothelial cells, patients with an HLA(A2)- mismatch with their donor were identified. HLA(A2) antigen was used in this study because it is one of the most common mismatching antigens among donor/recipients^11^. Cells from these patients were labelled with anti-human antibody HLA(A2)-BV421 (BD Bioscience) together with the other antibodies used to phenotype endothelial cells, except for CD133-BV421. Two patients without HLA-A2 were used as negative control for this patient subgroup.

### Biopsy examination

Biopsies were taken for cause and scored by a nephropathologist. Biopsies were scored as (a) borderline acute cellular rejection (ACR) if histology met the Banff 2019 lesion scores^42^ i ≥ 1 and t ≥ 1, (b) T-cell mediated rejection (TCMR) if histology met the Banff 2019 definition of TCMR (i.e. Banff lesion scores i ≥ 2 and t ≥ 2), or (c) acute Antibody mediated rejection (aABMR) if it met the Banff 2019 criteria for ABMR, including C4d- negative ABMR. Vascular damage was considered present when biopsies were classified as aABMR, TCMR2a, TCMR2b and TCMR3, following the Banff 2019 classification.

### Statistical and data analysis

All bioinformatics analyses were performed in R (version 3.6.2)^43^. Flow cytometry data was analyzed using the flowCore package^44^. Data was compensated using a spill over matrix generated with single labeled cells. Data quality was checked for anomalies regarding flow rate, signal acquisition and dynamic range using the *flow_auto_qc* function from flowAI^45^. Thresholds were set on FSC and SSC to remove non-single cells from the data. Fluorescence signal data were transformed to Logicle scale. Unbiased and unsupervised data clustering was performed using a non-linear generalization of principal component analysis (PCA) using flowSOM^46^. EC are a rare population, therefore, multiple clustering numbers and random seeds were used to determine the robustness of detected endothelial cell clusters between samples. Data set size and variance were also taken into consideration; 500 clusters were used for all patient data samples. Clusters were then displayed using t-SNE. A query was implemented to identify EC containing clusters and later those were manually inspected for all samples.

One-way ANOVA or Mann–Whitney U-test was used for statistical analysis wherever it was appropriate and described in the figure legend. A Bayesian Mann–Whitney U-test was also used to provide a more trustworthy perspective than the traditional frequentist analysis for the associations between cEC and rejection. Data shown are means ± SEM. P-value ≤ 0.05 was considered significant. Correction of p-values for multiple comparisons was done using the Benjamini and Hochberg procedure for controlling the false discovery rate (FDR).

## Supplementary Information

Supplementary Information.

## Data Availability

The data that support the findings of this study are available on request from the corresponding author.
