# Supplementary material for: Circulating endothelial cells transiently increase in peripheral blood after kidney transplantation
Source: Sci Rep. 2021 Apr 26;11:8915. doi: 10.1038/s41598-021-88411-4 (PMC8076225; doi:10.1038/s41598-021-88411-4)
Supplement: Supplementary file 1 — Supplementary Information. [file 41598_2021_88411_MOESM1_ESM.docx]

## **Supplementary Information.**

# **Circulating endothelial cells transiently increase in peripheral blood after kidney transplantation**

H. Tejeda-Mora, J.G.H.P. Verhoeven, W. Verschoor, K. Boer, D.A. Hesselink, M.W.F. van den Hoogen, L.J.W. van der Laan, C.C. Baan, R.C. Minnee, M.J. Hoogduijn


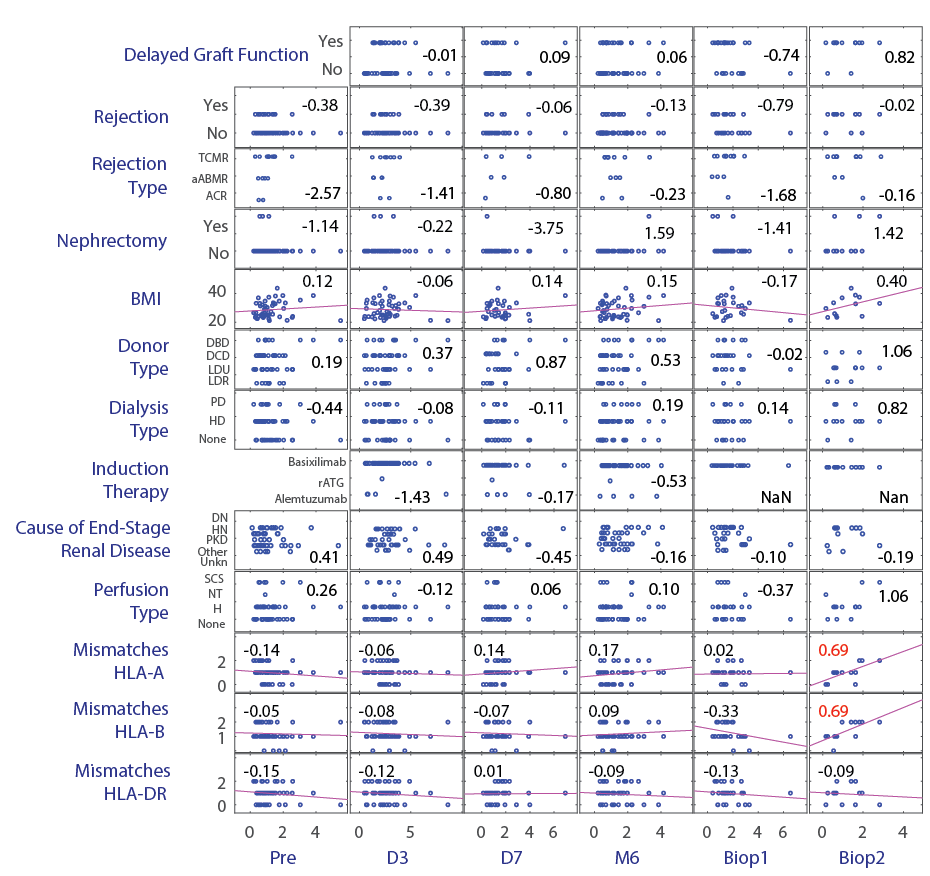


Supplementary Figure 1. Scatter plots with correlation between numbers of EC (x-axis) on measured time points and relevant clinical variables (y-axis). The displayed Pearson’s linear correlation coefficients indicate the slopes of the least-squares reference lines in the scatter plots. Logistic regression between numbers of EC in measured time points and relevant clinical variables. The displayed logistic coefficients indicate the change in the log odds of belonging to any of the observed variables. Coefficients marked in red are significant (p < 0.05). Abbreviations: Rejection type (TCMR: T cell Mediated Rejection, aABMR: acute Antibody-Mediated Rejection, Mixed Rejection); donor type (LRD: Living Related Donor, LURD: Living Unrelated Donor, DCD: Donation After Cardiac Death, DBD: Donation After Brain Death); dialysis type (H: Hemodialysis, PD: Peritoneal Dialysis); cause of end-stage renal disease (DN: Diabetic Nephropathy, HN: Hypertensive Nephropathy, PKD: Polycystic Kidney Disease, Unkn: unknown); perfusion type (HMP: Hypothermic Machine Perfusion, NMP: Normothermic Machine Perfusion, SCS: Static Cold Storage). Patients with DGF were such patients who needed renal replacement therapy within the first seven days after transplantation.


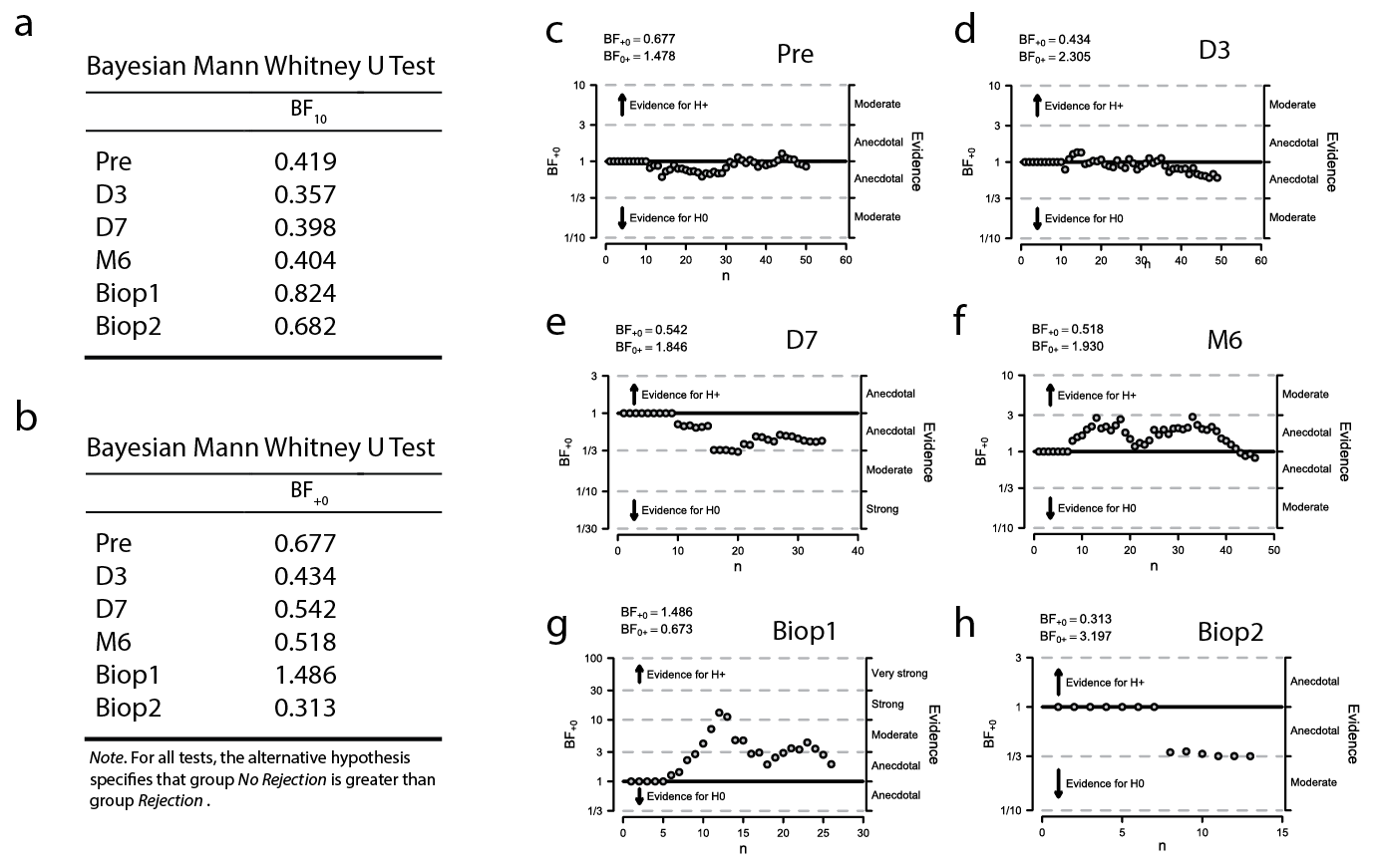


Supplementary Figure 2. Rejection and cEC number in kidney transplant rejectors. a) A two-sided analysis revealed a Bayes factor (BF_10_) that the data were ≤0.82 times more likely under the alternative (rejectors have higher cEC numbers) than the null hypothesis (rejectors and non-rejectors have similar cEC numbers). b) A subsequent one-sided test based on the alternative directional hypothesis that rejectors report a higher cEC number than non-rejectors (BF_+0_) resulted in a Bayes factor indicating that the data were ≤1.48 times more likely under this hypothesis than the null (Rejectors and non-rejectors have similar cEC numbers), with a median effect size of ≤0.49. c-h) Sequential analysis; dots account for all measurements at every timepoint. The evidence suggests that the data is best predicted by the null model and evidence for the alternative is inconsequential, therefore increasing patient inclusion is not encouraged.


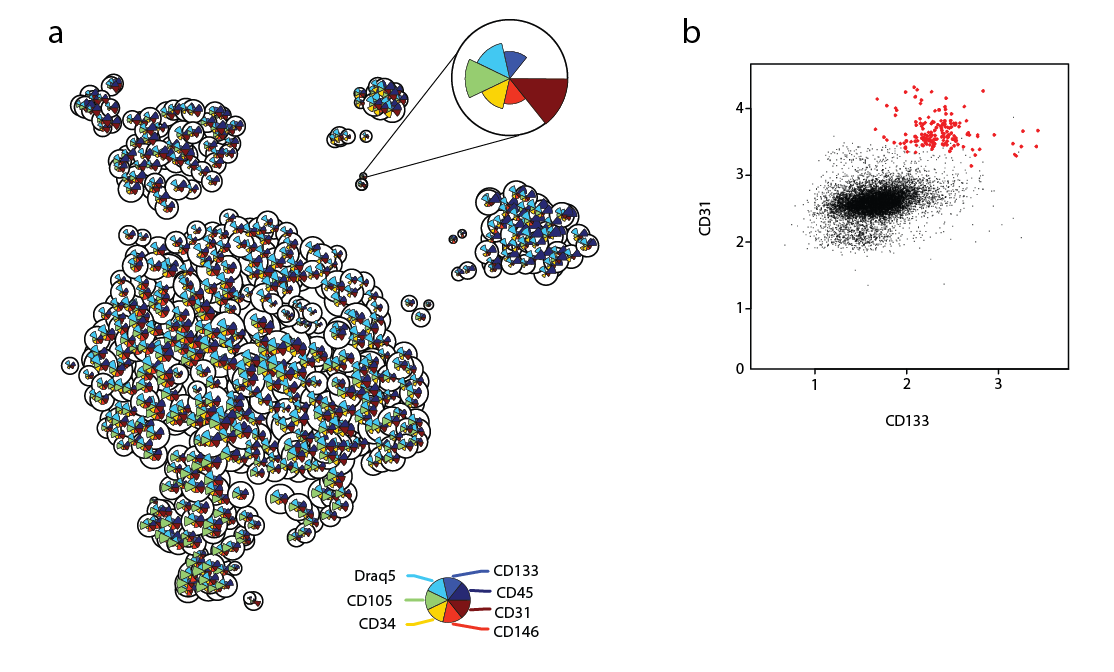


Supplementary Figure 3. Identification of EC in venous blood. Cells were stained for CD45, CD31, CD34, CD146, CD105, CD133, CD365 (not shown) and Draq5. cEC were identified by non-biased clustering. a) t-SNE representation of cell clusters; cluster size indicates amount of cells; pie charts inside clusters depict the mean fluorescence intensities (MFIs) of the indicated markers in the cluster. The EC cluster, containing 158 cells, is magnified. b) Panel showing the cEC cluster with markers CD31 and CD133.


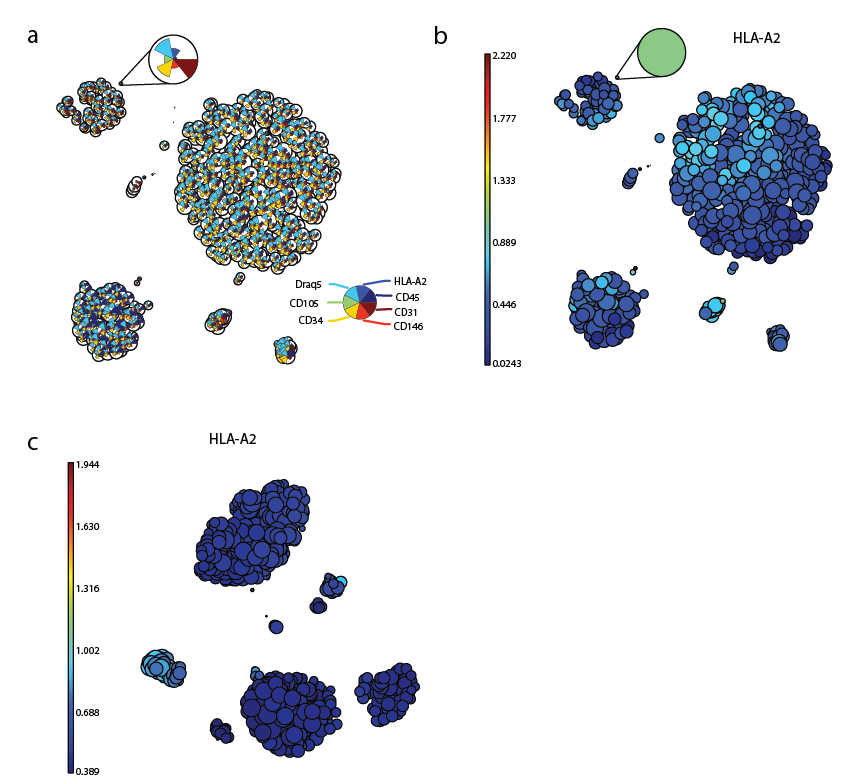


Supplementary Figure 4. Identification of HLA-A2 expressing cEC. Cells were stained for CD45, CD31, CD34, CD146, CD105, HLA-A2, CD365 (not shown) and Draq5 a-b) t-SNE representation of cell clusters from a kidney transplant recipient; only the donor expressed HLA-A2. Cluster size indicates amount of cells. c) t-SNE representation of cell clusters from a kidney transplant recipient (negative control). Both donor and recipient did not express HLA-A2. In a) pie charts inside clusters depict the mean fluorescence intensities (MFIs) of the indicated markers in the cluster; in b and c) expression of HLA-A2.


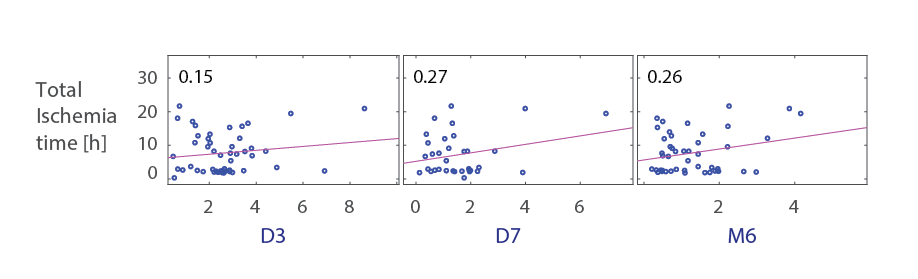


Supplementary Figure 5. Scatter plots with correlations between numbers of EC in measured time points and total ischemia time. The displayed Pearson’s linear correlation coefficients indicate the slopes of the least-squares reference lines in the scatter plots. Coefficients marked in red are significant (p < 0.05).

Supplementary Table 1. Timepoints and cEC numbers in patients with biopsy-proven rejection. Time point zero corresponds to transplantation day.

| **Patient** | **Sex** | **Rejection Type** | **Pre** | **D3** | **D7** | **Biop1** | **Biop2** | **Biop3** |
| --- | --- | --- | --- | --- | --- | --- | --- | --- |
| 1 | Male | aTCMR2B | - | 3; 1.46 | 5; 1.62 | 9; 1.88 | - | - |
| 2 | Female | borderline ACR | 0; 0.52 | 3; 2.87 | - | 6; 1.61 | 122; 1.95 | - |
| 3 | Male | aTCMR2A | 0; 1.10 | 4; 2.48 | - | 7; 1.81 | 81; 0.67 | 118; 0.86 |
| 4 | Male | caTCMR3 | -2; 0.75 | 3; 1.41 | - | 4; 041 | 10; 0.59 | - |
| 5 | Female | aABMR, C4d- | -1; 0.31 | - | 7; 1.68 | 4; 0.58 | 81; 070 | - |
| 6 | Male | aABMR, C4d+ | 0; 1.13 | 4; 3.30 | - | 7; 2.02 | 38; 1.82 | - |
| 7 | Female | aTCMR2A | -1; 1.36 | 2; 2.99 | 7; 3.90 | 5; 2.84 | - | - |
| 8 | Male | aTCMR1B + DN & aTCMR2A | -1; 1.40 | 4; 1.29 | - | 7; 2.04 | 39; 0.54 | - |
| 9 | Male | bTCMR | 0; 0.58 | 3; 2.04 | 6; 0.44 | 8; 0.80 | 14; 2.82 | - |
| 10 | Female | aTCMR2A & c-aTCMR2A | 0; 0.90 | 3; 2.20 | 7; 1.88 | 9; 1.29 | - | - |
| 11 | Female | aTCMR2A & aTCMR2A | 0; 1.51 | 3; 3.83 | - | 8; 1.32 | 15; 1.63 | 63; 1.60 |
| 12 | Female | aABMR, C4d- | -1; 1.08 | 4; 2.15 | - | 7; 0.84 | - | - |
| 13 | Male | aTCMR2A | -1; 2.55 | 3; 2.62 | - | 46; 1.45 | 95; 0.25 | - |
| 14 | Male | borderline ACR | 0; 0.79 | 3; 2.03 | 6; 0.38 | - | - | - |
| 15 | Female | aABMR, C4d- | 0; 0.49 | 2; 1.39 | - | 6; 0.82 | 115; 0.96 | - |

Supplementary Table 2. Demographic data of studied patients with HLA-A2 mismatch and their percentage of donor derived cEC.

| **Patient** | **Sex** | **Donor derived cEC %** | **Measured time points** | **HLA-A2 in donor** | **HLA-A2 in recipient** |
| --- | --- | --- | --- | --- | --- |
| 1 | Female | 0.95% | M6 | Yes | No |
| 2 | Female | 2.60% | M6 | Yes | No |
| 3 | Male | 1.70% | D3 | Yes | No |
| 4 | Male | 0.00% and 1.43% | Pre, D3 | Yes | No |
| 5 | Female | 3.50% | M6 | Yes | No |
| 6 | Female | 3.70% | M6 | Yes | No |
| 7 | Female | 1.36% | M6 | Yes | No |
| 8 | Female | 16.94% | D3 | No | Yes |
| 9 | Male | 4.69% and 1.82% | D3, day-10 biopsy | No | Yes |
| 10 | Male | 0.00% | D3 | No | Yes |
| 11 | Female | 17.28% and 18.91% | D3, D7 | No | Yes |
| 12 | Male | 4.62% | D3 | No | Yes |
| 13 | Male | 7.90% | M6 | No | Yes |
| 14 | Female | 1.15% | M6 | No | Yes |
| Control 1 | Female | 0.00% | M6 | No | No |
| Control 2 | Male | 0.00% | M6 | No | No |
